# Chain mediation of subjective assessment of insomnia and physical health in the relationship between loneliness and life satisfaction in undergraduate students: a biopsychosocial model of well-being

**DOI:** 10.3389/fpubh.2025.1730076

**Published:** 2026-01-23

**Authors:** Tahani K. Alshammari, Aleksandra M. Rogowska, Ghadah A. Alhussain, Sarah M. Alhatim, Basmah H. Alfageh, Haya M. Almalag, Musaad A. Alshammari

**Affiliations:** 1 Department of Pharmacology and Toxicology, College of Pharmacy, King Saud University, Riyadh, Saudi Arabia; 2 Institute of Psychology, Faculty of Social Sciences, University of Opole, Opole, Poland; 3 College of Pharmacy, King Saud University, Riyadh, Saudi Arabia; 4 Department of Clinical Pharmacy, College of Pharmacy, King Saud University, Riyadh, Saudi Arabia

**Keywords:** college students, insomnia disorder, life satisfaction, loneliness, mental health

## Abstract

**Introduction:**

University students are at risk of experiencing health and mental challenges, including loneliness, insomnia, and poor physical health, which can negatively impact their life satisfaction. Understanding the mechanisms underlying these factors’ association is essential for promoting students’ well-being.

**Methods:**

This is a cross-sectional survey-based study (from April to September 2024), involving 511 undergraduate students from several Saudi universities. We used validated measures of the General Health Scale, Satisfaction With Life Scale (SWLS), UCLA Loneliness Scale (ULS-8), and Athens Insomnia Scale (AIS). Sex differences were examined, and associations between loneliness, insomnia, physical health, and life satisfaction were analyzed using Pearson’s correlation and mediation models.

**Results:**

Our findings indicated that females reported significantly higher insomnia symptoms than males (*p* < 0.05). Life satisfaction was negatively correlated with loneliness, insomnia, and poor health (*p* < 0.001), while loneliness was positively associated with insomnia and poor health. Mediation analysis revealed that loneliness negatively contributes to life satisfaction both directly (*β* = −0.41) and indirectly through increased insomnia and poor physical health. Loneliness significantly reduces life satisfaction among university students, with insomnia and poor physical health partially mediating this relationship.

**Discussion:**

Addressing sleep disturbances and promoting physical health may help attenuate the adverse effects of loneliness and improve student well-being. Additionally, the results underscore the significance of social support in mitigating loneliness. Implementing future interventions, such as regular face-to-face interactions with friends and family, would help reduce loneliness and enhance life satisfaction.

## 1 Introduction

University and college students are particularly vulnerable to psychological distress, which can lead to the worsening or the onset of mental health conditions (1, 2). Mental health can be defined in two ways: as the absence of mental illness or as a broader concept that includes biological, psychological, and social factors influencing an individual’s well-being and ability to function (3). The Public Health Agency of Canada also describes it as the capacity to think, feel, and act in ways that foster resilience, enjoyment of life, and the ability to cope with challenges while recognizing the importance of culture, equity, social justice, personal dignity, and interconnectedness (3). Recent studies indicate that mental health challenges among university students are rising, with evidence suggesting an increase in distress and poor well-being over the past 5 years (1, 2). These issues not only pose a threat to students’ mental health but also have significant academic, social, and economic consequences (2). They can contribute to underperformance, a higher risk of insomnia and low sleep quality, reduced life satisfaction and social activity, and increased loneliness, ultimately affecting their overall well-being.

Insomnia is a sleep-related condition characterized by difficulty falling asleep, staying asleep, or experiencing non-restorative sleep despite having adequate opportunity to rest. To be diagnosed with insomnia, these sleep difficulties must occur and persist at least three times per week for at least 1 month and be associated with daytime impairment or distress (4). Among university students, insomnia is highly prevalent, with studies reporting rates ranging from 40 to 70% (5). The transition to university life introduces significant stressors, including academic workload, social changes, and irregular schedules, all of which contribute to sleep disturbances (2). Insomnia in students has been linked to impaired cognitive function, decreased academic performance, and increased risks of anxiety and depression (2, 5–7). In line with this, another study conducted in Greece during the COVID-19 pandemic reported that 65.9% of medical students had insomnia, with high levels of anxiety and depression being significantly associated with poor sleep quality (7).

Loneliness is a psychological and emotional state characterized by a sense of disconnection from others, often due to a lack of social belonging and meaningful relationships (8–10). Loneliness is a widespread and growing concern among university students, with significant implications for poor academic performance, as well as mental and physical health, including depression, anxiety, sleep problems, and increased risk of substance use (10–16). The transitions to university life, such as entering a new academic environment and facing social and academic pressures, have heightened the risk and prevalence of loneliness in this population (10, 11). The combination of academic pressure and social adjustment difficulties may lead to feelings of isolation, as students struggle to form meaningful connections in their new environment. Recent studies report that between 20 and 32% of university students experience loneliness, with some studies noting even higher rates during the COVID-19 pandemic (up to 76%) (10, 11, 14, 16–19). Key risk factors include being female or gender-diverse, younger or older age, being single, living alone, low socioeconomic status, poor health, low physical activity, and being a first-year or international student (10, 11, 13, 14, 16–20).

Furthermore, the experience of loneliness during these transitions can create a cyclical effect, where feelings of social disconnection reduce motivation to engage with peers or participate in campus activities, thereby reinforcing isolation. The lack of a stable social support system during such critical periods may exacerbate stress and hinder students’ ability to cope effectively with the demands of their new academic setting. Recognizing and addressing these vulnerabilities is essential for institutions aiming to promote student resilience and foster inclusive, supportive campus communities.

Loneliness is categorized into three primary forms: emotional, social, and existential. Both emotional (lack of close, intimate relationships) and social (lack of a broader social network) types of loneliness are prevalent among students (11, 21). Emotional loneliness arises when individuals lack close, meaningful relationships, often after losing significant people in their lives. Social loneliness occurs when individuals perceive their social network as inadequate in terms of both quantity and quality (10, 11, 22). A previous report has indicated that insomnia mediates health; it further documented that loneliness mediates the association between intergenerational relationships, as a reflection of quality of life in the aged population, and depression (23). In a previous study, loneliness correlated with lower life satisfaction among Polish, Spanish, and Slovak nursing students (22). Furthermore, loneliness was identified as a health and well-being indicator (24).

Life satisfaction is a key component of well-being, referring to an individual’s overall assessment of their life based on personal benchmarks and expectations. It encompasses both cognitive and emotional aspects, where individuals assess their circumstances in relation to their goals, desires, and past experiences (25, 26). As a broad indicator of quality of life and psychological health, life satisfaction significantly influences a person’s overall happiness and resilience. While psychological theories emphasize their importance, there is still much to uncover about the factors that shape it and its full impact on mental and emotional well-being (26, 27). A lower life satisfaction is often linked to hopelessness, which can elevate the risks of suicide. Thus, life satisfaction not only reflects overall well-being but also serves as a vital predictor of mental health outcomes (28, 29).

Beyond its connection to mental health, life satisfaction is shaped by factors such as academic experiences, social interactions, physical health, cultural influences, and individual characteristics (30, 31). Life satisfaction among university students is influenced by multiple factors, including socioeconomic status, academic performance, and quality of relationships with family and friends, with a majority reporting satisfaction in these areas (32). Cross-national studies among university students reveal that life satisfaction varies by country but consistently correlates with self-rated physical health, highlighting the universal importance of health perceptions (33). Academic stress is inversely related to life satisfaction, indicating that higher stress levels reduce students’ overall well-being (34). Additionally, cultural background and social support play crucial roles, with emotional and social health strongly predicting life satisfaction, emphasizing the need for university support systems tailored to diverse student populations (35, 36). During their studies, medical students may experience academic burnout, which can consequently reduce life satisfaction (37), whereas resilience helps counteract its effects and enhance well-being (38). Social influences, such as self-esteem and perceived discrimination, also play a role—self-esteem is particularly impactful in individualistic societies, whereas social belonging is more significant in collectivist cultures (25).

Physical health is another key determinant of well-being, with regular exercise linked to improved mood, reduced stress, and a greater sense of fulfillment (39). To understand university students’ physical health, it is essential to consider factors such as physical activity, diet, and sleep, and their impact on both physical and mental health. Although regular physical activity is crucial for maintaining physical health, university students often engage in low levels of physical activity, which is associated with poorer physical health outcomes (33, 40–44). For instance, a study in China found that 17.24% of students had low levels of physical activity, with a higher prevalence among females (44). Prolonged screen time and sedentary lifestyles also contribute to physical inactivity and mental health issues such as burnout and academic stress (41). Poor dietary habits, such as irregular breakfast consumption, low vegetable intake, and frequent consumption of sugar-sweetened beverages, are also prevalent issues that negatively impact students’ physical and mental health (45). Furthermore, irregular sleep patterns and poor sleep quality are common among university students and are associated with higher levels of stress, anxiety, and depression (41, 45, 46). Mediational analyses documented that life dissatisfaction and loneliness elevated the risks of depression, and these associations were mediated by general health (47).

A fundamental approach to understanding mental health is the biopsychosocial model (BPSM), which has been a key framework in psychiatric practice for four decades. First introduced by George Engel (48, 49). This model was developed as a response to the limitations of the traditional biomedical approach (50). At its core, the BPSM acknowledges that individuals experience illness as a whole rather than as isolated symptoms affecting specific organs. This model highlights the role of psychosocial factors in determining a person’s susceptibility to illness, symptom severity, and the overall progression of a disease (51).

This study aimed to investigate the relationship between life satisfaction and key factors such as loneliness, poor physical health, and insomnia symptoms in undergraduate students, controlling for sex as a biological key factor that has been documented to play a role in life satisfaction (52), and insomnia (53), where it prevailed in women compared to men. Gender bias was also reported to exist in general health (54). Therefore, we added biological sex as a possible confounding variable in our analysis. Furthermore, the interconnections among loneliness, poor physical health, and insomnia symptoms were investigated. Lastly, based on the biopsychosocial model of health and well-being, the study examined whether insomnia and physical health mediate the relationship between loneliness and life satisfaction, to provide a deeper understanding of the mechanisms linking these variables.

## 2 Methods

### 2.1 Study design and setting procedure

An observational, cross-sectional, survey-based study was conducted among undergraduate students in Saudi universities from April 24 to September 9, 2024. The sample size was determined using G*Power software (53). To test the mediation model, 119 participants were required, assuming a linear multiple regression analysis for life satisfaction as the dependent variable and four predictors (loneliness, insomnia symptoms, self-rated physical health, and biological sex), based on an *F*-test, fixed model, increased *R*^2^, with an alpha level of 0.05, desired power of 0.95, and a medium effect size (ƒ^2^ = 0.15). A minimum sample size of 176 participants (88 in each group) was required to detect a medium effect (*d* = 0.5, *α* = 0.05, 0.95 power) in a Student’s *t*-test with two independent groups (female and male university students).

Data were collected via a convenient sampling technique using validated Arabic and English versions of the surveys. The survey was distributed online via social media and on-campus visits. The online distribution was conducted via social media platforms such as Twitter, WhatsApp, and LinkedIn, targeting universities across Saudi Arabia. Additionally, visits were made to both male and female university campuses at KSU to gather survey data. Participation was entirely voluntary, and they provided their consent to complete the survey.

The initial version of the survey was piloted and distributed to a sample of 12 individuals. Each participant was asked to complete the survey and then provide feedback on the clarity, relevance, and overall structure of the questions. Ethical approval was granted by the Institutional Review Board at King Saud University in Riyadh, Saudi Arabia (Ref. No. 24/1107/IRB 23, issued on February 13, 2024). Informed consent was obtained from all participants involved in the study.

### 2.2 Participants’ characteristics

The survey was distributed to adult students attending universities in Saudi Arabia. Our study questionnaire included demographic information such as age (categorized by year range), biological sex, marital status, study year, and specialty track. A total of 511 students participated in the study, comprising 72.60% females and 98.24% unmarried participants (Table 1). Most of the students were between 18 and 23 years old (90.02%), studying the health track (70.06%), with the majority being in their second year of studies (31.51%), which is commonly referred to as the first year of specialty after the preparatory year. The sample size was more than appropriate for the study.

**Table 1:** Participant characteristics (*N* = 511).

Variable	Categories	n/M	%/*SD*
Age	18–20	241	47.16
	21–23	219	42.86
	24–26	43	8.42
	Above 26	8	1.57
Gender	Female	371	72.60
	Male	140	27.40
Marital status	Married	9	1.76
	Single	502	98.24
Study track	Health track	358	70.06
	Humanities track	41	8.02
	Science track	109	21.33
	Technical	3	0.59
Study year	1 year	41	8.02
	2 year	161	31.51
	3 year	83	16.24
	4 year	78	15.26
	5 year	67	13.11
	6 year	34	6.65
	Internship	47	9.20
Loneliness (UCLA-8)	Total score (8–31)	17.24	5.15
Physical health (GSRH-2)	Total score (2–10)	4.28	1.87
Insomnia (AIS-8)	Total score (0–21)	7.69	4.17
	Absence of insomnia	176	34.44
	Mild insomnia	172	33.66
	Moderate insomnia	137	26.81
	Severe insomnia	26	5.09
Life satisfaction (SWLS-5)	Total score (5–35)	25.46	6.17
	Extremely dissatisfied	9	1.76
	Dissatisfied	21	4.11
	Slightly dissatisfied	53	10.37
	Neutral	19	3.72
	Slightly satisfied	125	24.46
	Satisfied	180	35.23
	Extremely satisfied	104	20.35

### 2.3 Measures

#### 2.3.1 Loneliness

The UCLA-8 Loneliness Scale (54, 55) contains eight items, for example, item 3: “I am an outgoing person,” and Item 6: “I can find companionship when I want it.” Each item has a 4-level frequency score, with answer choices of 1 (never), 2 (rarely), 3 (sometimes), and 4 (always). The higher total score suggests a higher degree of loneliness. The reliability of the UCAL-8 was assessed using Cronbach’s *α*, which was 0.80 in the present study sample.

#### 2.3.2 Physical health

The General Self-Rated Health (GSRH) (56–58) was used in this study to evaluate health-related quality of life. As a concise alternative to the standard general health survey (SF-12 V), the GSRH consists of two single-item questions. The first question (GSRH-1) was “In general, would you say your health is …?” and the second question (GSRH −2) was “Compared to others your age, would you say your health is …?” and asks participants to rate their response on a five-point Likert scale, ranging from 1 (Excellent) to 5 (Poor). The internal consistency of GRSH-2 in this study was Cronbach’s *α* = 0.82.

#### 2.3.3 Insomnia

The Athens Insomnia Scale (AIS) is a self-administered questionnaire designed to assess sleep difficulties, particularly insomnia (59, 60). It consists of eight items, for example, an item is “Sleep induction (time it takes you to fall asleep after turning off the lights), each rated on a Likert scale from 0 to 3. Higher scores reflect more severe insomnia symptoms. In this study, the reliability of AIS-8 was adequate (Cronbach’s *α* = 0.78).

#### 2.3.4 Life satisfaction

The Satisfaction With Life Scale (SWLS) is a five-item measure developed by (61, 62) to assess individuals’ overall cognitive judgments of their life satisfaction. Participants rate their agreement with each statement on a 7-point scale, ranging from 1 (Strongly Disagree) to 7 (Strongly Agree). The SWLS had high reliability in this study (Cronbach’s *α* = 0.85).

### 2.4 Statistical analysis

Descriptive statistics, including mean (M), standard deviation (SD), and median (Mdn), were used to describe the study variables. Since the study sample was large (*N* = 511) and skewness and kurtosis were within ±1, we assumed the sample did not deviate significantly from normality; therefore, we used parametric statistics to test the research questions. Sex differences were compared on loneliness, physical health, insomnia symptoms, and life satisfaction using an independent-samples Student’s *t*-test. The associations between loneliness, physical health, insomnia symptoms, and life satisfaction were examined using Pearson’s correlation analysis. A multiple linear regression model was performed, with life satisfaction as the dependent variable and loneliness, physical health, and insomnia as independent variables, while controlling for biological sex as a potential confounder. The mediating roles of insomnia and physical health in the relationship between loneliness and life satisfaction were tested using the Classical Process Model, which used the PROCESS macro (63, 64) to estimate mediation. The Model 6 was used for this study, in which insomnia (M1) and physical health (M2) were treated as chain mediators in the relationships between loneliness (X) and life satisfaction (Y). The Classical Process Model is a flexible, regression-based framework for analyzing complex causal pathways, allowing researchers to test indirect effects and gain a more nuanced understanding of how variables work together, going beyond simple X-to-Y relationships. All statistical analyses were performed using JASP version. 0.19.3.0 for Windows (65).

## 3 Results

### 3.1 Sex differences in loneliness, physical health, insomnia symptoms, and life satisfaction

Significant sex differences have been found for insomnia symptoms. Females scored higher than males in insomnia symptoms, but the effect size was small (*p* < 0.05, Cohen’s *d* = 0.23). However, there was no statistically significant difference between females and males in loneliness, physical health, and life satisfaction (Table 2).

**Table 2:** Student’s *t*-test for loneliness, physical health, insomnia symptoms, and life satisfaction across genders (*N* = 511).

Variable	Female (*n* = 371)		Male (*n* = 140)		*t*(509)	*p*	*d*
	*M*	*SD*	*M*	*SD*			
Loneliness	17.49	5.08	16.59	5.28	1.75	0.080	0.17
Insomnia	7.95	4.19	7.01	4.07	2.27	0.024	0.23
Physical health	4.35	1.89	4.09	1.81	1.44	0.150	0.14
Life satisfaction	25.40	6.07	25.60	6.44	−0.32	0.749	−0.03

### 3.2 Associations between loneliness, physical health, insomnia symptoms, and life satisfaction

**Figure 1:**
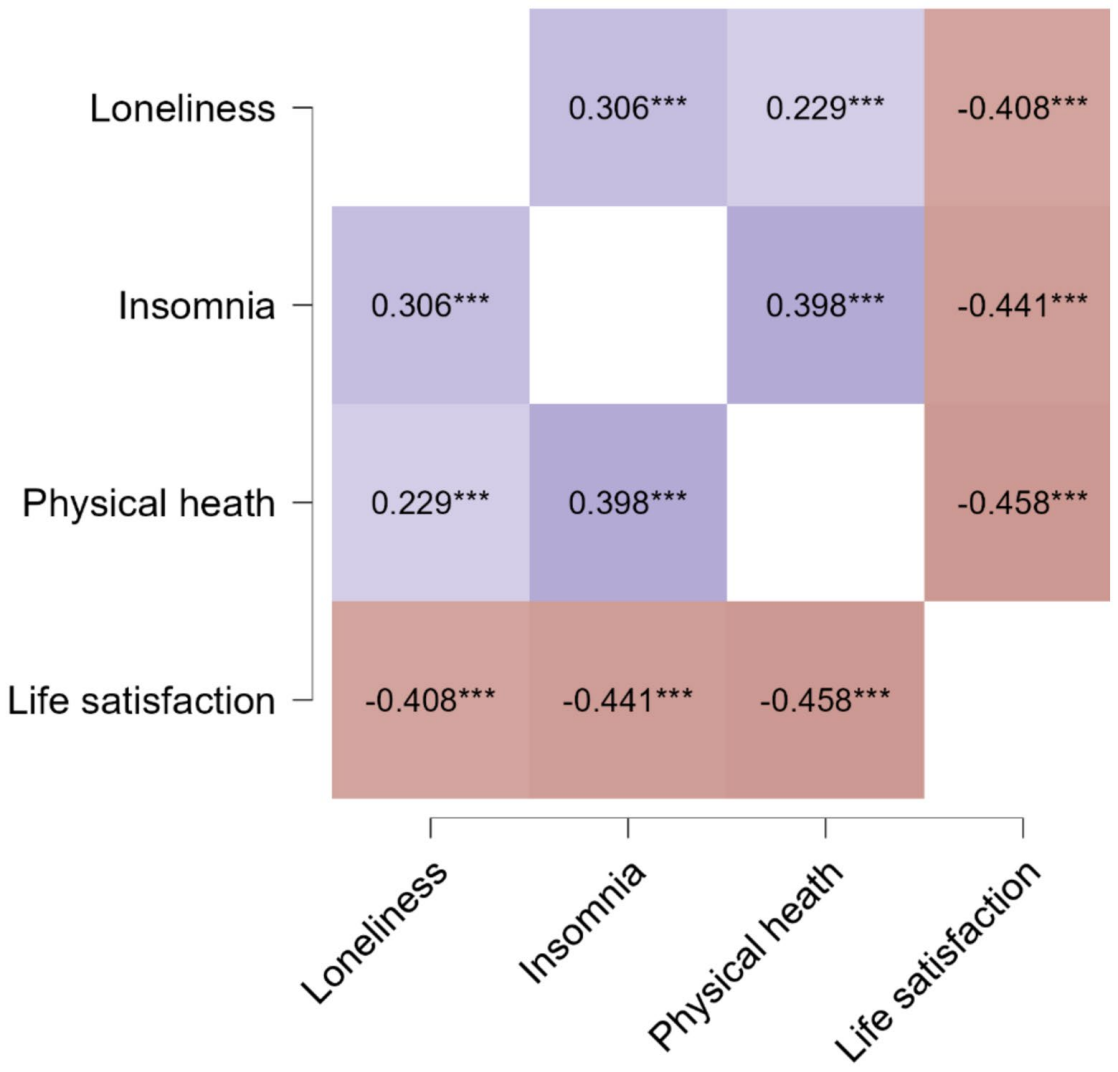
Pearson’s *r* heatmap for associations between loneliness, poor physical health, insomnia symptoms, and life satisfaction (*N* = 511). ***p < 0.001.

The correlation matrix is presented in Figure 1. All variables were interrelated, with small effect sizes (r ranged from 0.229 to −0.458) and high levels of significance (*p* < 0.001). As expected, life satisfaction was negatively related to loneliness, insomnia symptoms, and poor health. Positive correlations were found between loneliness, insomnia symptoms, and poor health.

### 3.3 Mediation model

The analysis of the associations between life satisfaction (the explained variable) and loneliness, insomnia symptoms, and physical health (the predictors), while controlling for biological sex as a confounder (Table 3). The regression model M_0_ explained 17% of the life satisfaction variance and included biological sex (coded Female = 1, male = 0), together with loneliness (R = 0.41, R^2^ = 0.17, *F*(2, 508) = 50.88, *p* < 0.001). Loneliness was a significant predictor of life satisfaction (*β* = −0.41), while biological sex was not significantly related to life satisfaction. All variables in model M1, including loneliness, insomnia symptoms, and poor physical health, were negative predictors of life satisfaction. The regression model M_1_ explained 36% of the variance in life satisfaction, *R* = 0.60, *R*^2^ = 0.36, *F*(2, 506) = 69.54, *p* < 0.001.

**Table 3:** Multiple linear regression for life satisfaction (*N* = 511).

Model		*b*	*SE*	β	*t*	*p*	95% CI		Collinearity	
							Lower	Upper	Tolerance	VIF
M₀	(Intercept)	33.98	0.90		49.08	< 0.001	24.58	26.63		
	Sex	−0.24	0.56		−0.32	0.749	−1.40	1.01	0.99	1.01
	Loneliness	−0.49	0.05	−0.41	−10.08	< 0.001	−0.59	−0.40	0.99	1.01
M₁	(Intercept)	38.23	0.87		43.78	< 0.001	36.51	39.94		
	Sex	0.69	0.50		1.40	0.163	−0.28	1.67	0.99	1.01
	Loneliness	−0.32	0.05	−0.27	−7.09	< 0.001	−0.41	−0.23	0.89	1.12
	Physical health	−1.00	0.13	−0.30	−7.72	< 0.001	−1.25	−0.74	0.83	1.21
	Insomnia	−0.36	0.06	−0.24	−6.05	< 0.001	−0.48	−0.24	0.79	1.27

CI, confidence interval. Female sex was included in the regression model (biological sex coded: Female = 1, male = 0).

**Figure 2:**
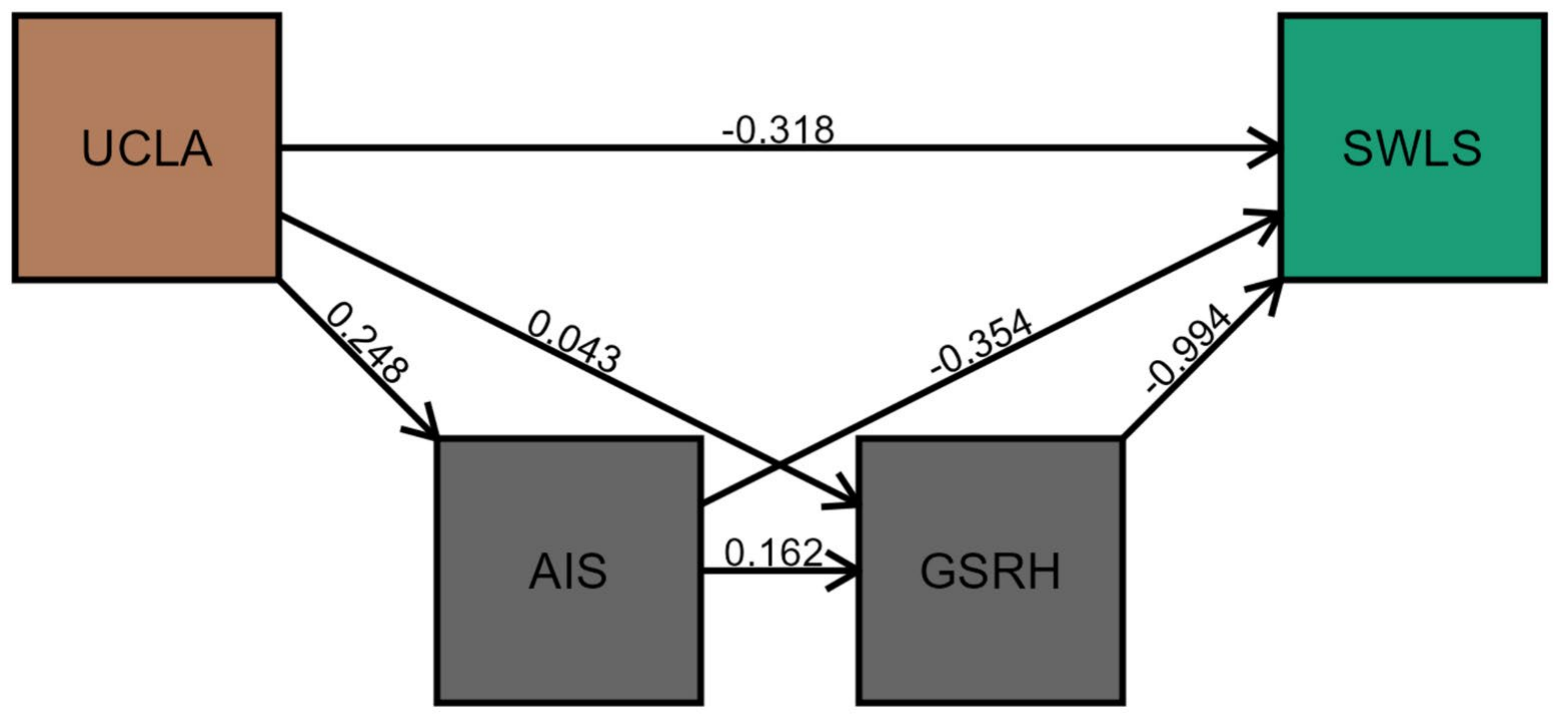
Chain mediation effects of insomnia (AIS) and poor physical health (GSRH) on the relationship between loneliness (UCLA) and life satisfaction (SWLS). Estimates in the figure are unstandardized regression coefficients (*b*).

The mediating effects of insomnia and physical health on the relationship between loneliness and life satisfaction were examined using the classical process model (63, 64). All paths were significant for life satisfaction, suggesting partial mediation effects (Figure 2). Loneliness is negatively related to life satisfaction (standardized total effect estimate: *β* = −0.41). When insomnia and poor physical health were included in the regression model, the relationship between loneliness and life satisfaction became weaker (standardized direct effect: β = −0.27). The chain mediation model explains 62% of the variance in life satisfaction.

## 4 Discussion

In this study, we examined the intricate relationships among loneliness, insomnia symptoms, physical health, and life satisfaction, while accounting for confounding variables such as biological sex, within a biopsychosocial framework. Our results revealed that females reported more pronounced insomnia symptoms than males. Life satisfaction declined as loneliness, insomnia, and poor physical health increased, suggesting that these factors are negatively correlated. Additionally, loneliness showed positive associations with both insomnia symptoms and poor physical health. Furthermore, our mediation analysis showed that loneliness reduced life satisfaction both directly and indirectly, by increasing insomnia symptoms and deteriorating physical health. These findings collectively underscore the mediating roles of insomnia and health status in the relationship between loneliness and life satisfaction, supporting the relevance of the biopsychosocial model in understanding student mental health.

Our study found no statistically significant differences between male and female participants in loneliness, physical health, and life satisfaction, aligning with previous research indicating that biological sex does not substantially influence overall life satisfaction. For example, an earlier study found that women reported higher levels of life satisfaction than men; however, these differences were inconsistent across age groups (52). Furthermore, Jordan et al. (66) found no significant sex differences in life satisfaction, social support, or self-esteem among undergraduate students, though Females exhibited higher gratitude levels than males (66). Regarding physical health, gender differences were reported to be a condition-based status, where it was found that men presented higher risks in some disorders. In comparison, women tend to have higher risks in other conditions (54).

Similarly, Froh et al. (67) demonstrated that the relationship between gratitude and life satisfaction did not significantly differ by gender, suggesting a shared underlying mechanism between males and females. These findings indicate that while some psychological traits may vary by biological sex and psychosocial gender, life satisfaction appears to be influenced by factors beyond sex alone. Yet, our findings suggest that insomnia symptoms vary across biological sex.

At the same time, Tang et al. (68) supported our findings regarding insomnia results. They reported that females had more sleep difficulties than males, including longer sleep latency and poorer sleep quality (68). It further supported our previous reports indicating that insomnia rates are higher in student samples composed primarily of females compared to the global prevalence (69, 70).

In line with our findings, a systematic review found that approximately 30% of university students met criteria for insomnia, and poor sleep was directly linked to academic stress and reduced performance (6). Additionally, we previously reported that a significant number of undergraduate students exhibited insomnia at higher rates (69, 70). Furthermore, another study reported a negative correlation between physical activity and insomnia, suggesting that physically active students reported fewer sleep problems (5).

Loneliness negatively correlates with life satisfaction, demonstrating its detrimental effects on well-being (71). A study on a Chinese university student sample revealed that individuals with high scores of loneliness are more prone to mood disorders, including anxiety, depression, and unhealthy coping mechanisms. Furthermore, prolonged loneliness increases the risk of long-term psychological distress, with lonely youth being three times more likely to develop depression later in life (72). In line with this, a study among health track students found that participants with higher levels of loneliness reported lower satisfaction across various aspects of life, reinforcing the idea that loneliness significantly affects mental and emotional well-being (22).

Loneliness directly reduces life satisfaction, and this is partially due to increased insomnia symptoms, which, in turn, lead to lower subjective assessments of physical health, ultimately resulting in lower life satisfaction. These results align with the biopsychosocial model of health and well-being, which highlights the interplay between psychological, physiological, and social factors in shaping individuals’ quality of life (73).

Our analysis demonstrated that insomnia symptoms and poor physical health mediate the relationship between loneliness and life satisfaction. Accumulating evidence indicates that loneliness significantly correlates with sleep disturbances, negatively affecting overall well-being (74). Hom et al. (75) found that severe insomnia symptoms were associated with greater loneliness and predicted higher loneliness levels (75). Similarly, Gu et al. (76) demonstrated that loneliness was linked to sleep disturbances, which leads to reduced mental well-being. Their findings identified insomnia as a mediator that amplifies loneliness’s negative consequences, ultimately decreasing life satisfaction. Additionally, a previous report found that loneliness was associated with poor physical health outcomes such as stroke, angina, and increased difficulty in daily activities, all of which contribute to lower life satisfaction (77).

Consistent with previous research, our study confirmed that loneliness has a direct negative impact on life satisfaction, supporting the idea that increased loneliness corresponds with lower subjective well-being. Deutrom et al. (78) demonstrated a negative association between loneliness and life satisfaction. Similarly, Kupcewicz et al. (22) found strong negative correlations between loneliness and life satisfaction among nursing students across multiple countries, highlighting the universal nature of this relationship.

This study is the first of its kind exploring this interaction among university students in Saudi Arabia. Cultural differences can exist between Saudi students and those from other countries, as suggested by research (79, 80). These differences primarily arise from Saudi Arabia’s deeply conservative, Islamic-influenced culture, which significantly impacts lifestyle. It includes a firm reliance on family and relationships, gender segregation in education and religion, and limited male–female social interaction and communication. Such cultural characteristics often present challenges for international students who adhere to more liberal norms. Saudi Arabian culture is predominantly characterized by collectivism, emphasizing group harmony, loyalty, and the importance of peer recommendations. It stands in contrast to more individualistic cultures, where personal achievement is prioritized. Islam plays a significant role in shaping daily life, social norms, and lifestyle, which can differ markedly from secular or minority-faith contexts, influencing aspects such as dress codes and daily routines. Although Arabic is the primary language, there are notable differences in non-verbal communication, such as eye contact and gestures, as well as in communication styles, necessitating adaptation, particularly for non-Arab students. While some Saudi students exhibit a positive disposition towards Western culture due to educational requirements, such as the need for English proficiency, deeply ingrained cultural values continue to influence their perceptions and interactions (79, 80). This work sheds more light on the similarities and differences between Saudi students and those from other cultural and religious backgrounds, as documented in the literature. Understanding provides better opportunities to support students in this critical phase of their lives.

Several limitations were observed in this study. Culture and religion, among other factors, need to be explored in depth to examine their relationships with insomnia, physical health, and life satisfaction. Future studies with a qualitative design are needed to investigate the impact of these factors. This study presents several noteworthy limitations that should be addressed in future research to strengthen the validity and generalizability of the findings. Primarily, the cross-sectional design restricts the ability to draw causal conclusions regarding the relationships among loneliness, insomnia, physical health, and life satisfaction. While significant associations were identified, the temporal sequence and causal pathways implied by the chain mediation model remain speculative. Longitudinal or experimental designs are essential for confirming these directional relationships and for better understanding how changes in one variable may influence others over time.

Additionally, the reliance on self-reported data introduces potential biases such as social desirability, recall inaccuracies, and response bias, which may affect the accuracy of the measured variables. Incorporating objective measures—such as medical records to verify physical health status or actigraphy to assess sleep patterns—would enhance data precision and reduce subjective bias. Furthermore, critical contextual factors such as culture and religion were not explored in depth, yet they may play a significant role in shaping experiences of insomnia, health, and life satisfaction. Qualitative studies are recommended to capture these complex influences and provide richer insights into how sociocultural dimensions interact with the variables under study, thereby offering a more comprehensive understanding of the phenomena under investigation.

## 5 Conclusion

In conclusion, our study underscores the importance of addressing loneliness and its associated health consequences to enhance life satisfaction among university students. The findings underscore the importance of targeted interventions that promote social connectedness, enhance sleep quality, especially in females, and promote physical well-being, ultimately contributing to a more comprehensive approach to student health and well-being.

## Data Availability

The datasets presented in this study can be found in online repositories. The names of the repository/repositories and accession number(s) can be found at: the data presented in this study are openly—available in Mendeley Data at 10.17632/9xf92fry3v.1 reference number.
